# Longitudinal exposure to antiseizure medications shape gut-derived microbiome, resistome, and metabolome landscape

**DOI:** 10.1093/ismeco/ycae123

**Published:** 2024-10-18

**Authors:** Camille Dop, Stéphane Auvin, Stanislas Mondot, Patricia Lepage, Zehra Esra Ilhan

**Affiliations:** Université Paris-Saclay, Institut National de Recherche pour l’Agriculture, l’Alimentation et l’Environnement (INRAE), AgroParisTech, Micalis Institute, Domaine de Vilvert, Jouy-en-Josas, France; Université de Paris, Institut National de la Santé et de la Recherche Médicale (INSERM), NeuroDiderot, Paris, France; Pediatric Neurology Department, Assistance Publique-Hôpitaux de Paris (APHP), Robert Debré University Hospital, Paris, France; Institut Universitaire de France, Paris, France; Université Paris-Saclay, Institut National de Recherche pour l’Agriculture, l’Alimentation et l’Environnement (INRAE), AgroParisTech, Micalis Institute, Domaine de Vilvert, Jouy-en-Josas, France; Université Paris-Saclay, Institut National de Recherche pour l’Agriculture, l’Alimentation et l’Environnement (INRAE), AgroParisTech, Micalis Institute, Domaine de Vilvert, Jouy-en-Josas, France; Université Paris-Saclay, Institut National de Recherche pour l’Agriculture, l’Alimentation et l’Environnement (INRAE), AgroParisTech, Micalis Institute, Domaine de Vilvert, Jouy-en-Josas, France

**Keywords:** microbiota, anti-epileptic drugs, glutamine, chronic exposure, antimicrobial resistance genes, exposome, infants, xenobiotics, multiomics

## Abstract

The influence of chronically administered host-targeted drugs on the gut microbiome remains less understood compared to antibiotics. We investigated repetitive exposure effects of three common antiseizure medications [carbamazepine (CBZ), valproic acid, and levetiracetam] on the gut microbial composition, resistome, and metabolome using microcosms constructed from feces of young children. Microcosms were established by cultivating feces for 24 h (C0). These microcosms were daily transferred into fresh media for seven cycles (C1–C7) with antiseizure medications or carrier molecules, followed by four cycles without any drugs (C8–C11). The microbial dynamics and resistome of microcosms at C0, C1, C7, and C11 were assessed with 16S ribosomal ribonucleic acid gene sequencing or shotgun metagenome sequencing and real-time quantitative polymerase chain reaction analysis of the antimicrobial resistance genes, respectively. Metabolites of CBZ-treated and control microcosms at C0, C1, and C7 were evaluated using non-targeted metabolomics. Our findings revealed that the serial transfer approach longitudinally altered the microcosm composition. Among the medications, CBZ had the most substantial impact on the structure and metabolism of the feces-derived microcosms. The microbiome composition partially recovered during the drug-free period. Specifically, *Bacteroides* and *Flavonifractor* were depleted and *Escherichia* and *Clostridium* were enriched. Additionally, repetitive CBZ exposure increased the abundance and expression of genes related to various antibiotic resistance mechanisms, more specifically, efflux pumps and antibiotic target alteration. CBZ-induced changes in the microbiome were mirrored in the metabolome, with reductions in the citric acid cycle metabolites, glutamine, and spermidine, alongside increased levels of vitamin B6. Our study suggests that repetitive CBZ exposure may negatively impact gut microbial homeostasis and metabolism.

## Graphical Abstract

**Graphical Abstract:**
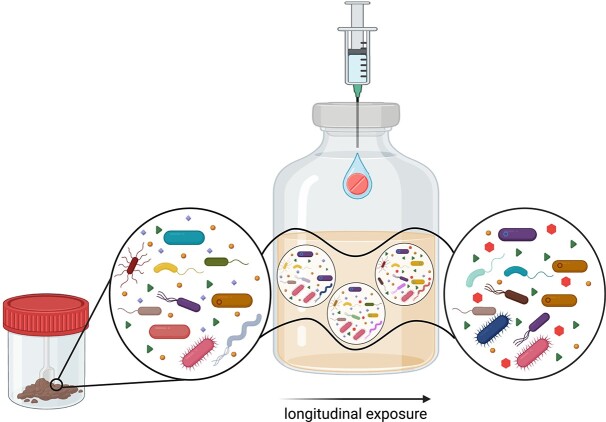

## Introduction

Epilepsy is a common neurological disorder globally affecting ~50 million people (World Health Organization, February 2024) [1]. It often appears during childhood, and epileptic seizures has life-long developmental consequences. Today, there is no cure for epilepsy, but its symptoms can be managed by chronic use of antiseizure medications (ASMs). Currently, there are >20 ASMs available, including carbamazepine (CBZ), valproic acid (VALP), and levetiracetam (LEV) [2]. Even though these medications are largely considered safe, long-term daily use and cumulative dose effects have been associated with adverse health events including vascular diseases and osteoporosis [3, 4].

The ASMs may negatively impact the gut microbiome, collectively, microorganisms, their genes, and metabolites. The gut microbiome in early life is especially vulnerable to drugs as it is established during the first 3 years of life. For example, in children, antibiotics induce dysbiosis in the gut microbiota by reducing its diversity [5], which is associated with an increased risk of metabolic or inflammatory diseases [6, 7]. Additionally, depletion of the gut microbiome due to long-term antibiotic therapy altered bile and fatty acid metabolisms in rats [8]. Host-targeted drugs may also exert anti-commensal effects on the gut microbiome [6, 7]. We recently demonstrated that several ASMs, including CBZ and VALP, inhibited the growth of several early-life associated gut bacterial species in monocultures [9]. However, microbial response could be different in communities than in monocultures. For example, prolonged exposure to 5 g/kg/day of valproate enhanced the diversity of the microbiome and modulated the fecal concentration of various short-chain fatty acids (SCFAs) in rats [10]. While the majority of the studies on host-targeted drugs and gut microbiome interactions are based on single exposure studies [9, 11, 12], the cumulative impact of these long-term therapies on the microbial community dynamics and metabolic interactions are often neglected.

Drug-induced alterations in the gut microbiome structure may also have implications for the spread of antimicrobial resistance genes (ARGs). Antibiotics and host-targeted drugs including psychotropic drugs can increase antibiotic resistance in the environment [13–15]. For example, CBZ may increase the likelihood of ARG spread by enhancing membrane permeability and subsequently facilitating plasmid transfer among the bacteria [14]. Although these studies were performed in environmental ecosystems, the complexity of the communities and the selective pressure exerted on these microorganisms are similar to those found in the human gut microbiome. Furthermore, a recent zebrafish study also showed that chronic CBZ exposure modulated gut microbial diversity and abundance of ARGs [16].

The chronic exposure effects of ASMs on complex early-life gut microbiome structure, abundance of ARGs, and metabolic function is still poorly understood. In this study, we investigated the impact of repetitive exposure to three ASMs (CBZ, VALP, and LEV) on microbial community dynamics, expression of ARGs, and metabolome. We used a serial transfer approach, exposing feces-derived microbial communities to the ASMs for 7 days followed by a drug-free period of 4 days. Our findings elucidate that chronic exposure to CBZ disrupts gut microbial dynamics with partial recovery observed upon its removal, increases expression of ARGs, and ultimately negatively impacting amino acid, energy, polyamine, and vitamin B6 metabolisms.

## Materials and methods

### Sample collection and ethics statement

Microcosms were constructed from the feces (A, B, C, and D) of four children (11 months to 5 years, 2 females and 2 males) with no apparent health condition or medication use at least 2 months before the sample collection. Immediately after defecation, parents collected ~1 g of feces into a stool collection tube, sealed it in an airtight bag with an anaerobic atmosphere generator (bioMérieux, Paris, France), and stored at 4°C for 4–6 h. The French National Ethics Committee for the Protection of People (Comité de Protection des Personnes) approved fecal sample collection. The legal representatives of the children provided informed consent and collected fecal samples.

### Inoculum preparation

The fecal samples were homogenized in 10 mL of modified Gifu anaerobic medium (mGAM, NISSUI Pharmaceutical, Tokyo, Japan) with sterile glass beads by vortexing for 10 min at 2500 rpm in an anaerobic chamber (Bactron 600, Sheldon Manufacturing Inc., Cornelius, Oregon, United States). Fecal slurries were mixed with glycerol (final concentration of 25%) and stored at −80°C.

### Chemicals

The ASM active ingredients (CBZ, VALP, and LEV) and all media supplies were purchased from Sigma Aldrich and Merck (Darmstadt, Germany). The 220 mM stock solutions of CBZ were prepared in dimethyl sulfoxide (DMSO) and other compounds in sterile water and kept at 4°C.

### Microcosm experiments

To establish the microcosms, 0.5 mL of fecal slurries were inoculated into 40 mL serum bottles containing 20 mL of anaerobic mGAM supplemented with 30 mM NaHCO_3_^−^ and 0.01% L-cysteine. The cultures were incubated at 37°C with shaking at 80 rpm for 24 h.

**Figure 1:**
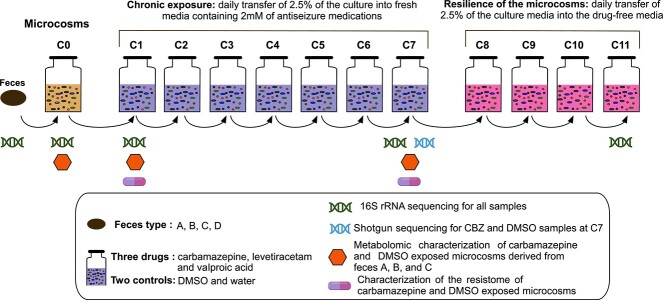
Study design and establishment of semi-continuous microcosms. The experiment design relied on the daily transfer of 2.5% of feces-derived microbial cultures (C0 microcosms) into fresh mGAM media for 11 cycles (C1–C11). The microcosms were daily exposed to ASMs for seven cycles (C1–C7) followed by four drug-free cycles (C8–C11). Microbiome dynamics were characterized for C0, C1, C7, and C11 based on 16S rRNA gene sequencing. The metabolome of CBZ and DMSO (DMSO-Ctrl) exposed microcosms (A, B, and C replicates) were characterized for cycles C0, C1, and C7. The metagenomes of CBZ and DMSO-Ctrl were characterized through shotgun sequencing for the C7 cycle. The resistome of CBZ and DMSO-Ctrl were characterized for cycles C1 and C7.

The 24-h cultures were considered as cycle 0 (C0) and used as inoculum for ASM exposure assays. As shown in Fig. 1, we utilized a semi-continuous system that relied on daily transfer of the cultures (2,5%) into fresh anaerobic media (20 mL) with either one of the ASMs (CBZ, LEV, or VALP) or drug carrier molecules (water or DMSO) for 7 days (cycles C1–C7) followed by transfers into drug-free media (cycles C8–C11) for 4 days. This approach allowed us to longitudinally monitor gut microbiome dynamics including adaptation and resilience. The 2 mM concentration was selected based on the average estimated concentration of the three ASMs administered daily to children and their observable impact on microbial growth [9]. The final DMSO concentration of <1% was similar to the values typically used in drug screening assays [11]. All experiments were performed with four biological and two technical replicates. All inoculations and samplings were performed in the anaerobic chamber.

The pH measurements (ranged from 6.4–6.9) were done with a pHmeter (©Mettler-Toledo, Viroflay, France). At the end of each cycle, three 1.8 mL samples were taken from each serum bottle and centrifuged at 21 300 g for 3 min to separate pellets for nucleic acid extraction and supernatants (filtered through Millex®-GV, Hydrophilic Durapore® 0.22 μm polyvinylidene fluoride membrane) for metabolome analysis. The samples were stored at −80°C.

### Deoxyribonucleic acid and ribonucleic acid extractions

Deoxyribonucleic acid (DNA) was extracted from the feces and the microcosm pellets at C0, C1, C7, and C11 using the Qiamp®PowerFecal kit (Qiagen, Hilden, Germany), resuspended in 75 μL of Tris-ethylenediaminetetraacetic acid buffer and stored at −20°C. DNA quality and concentrations were determined using Nanodrop (Thermo Fisher Scientific, Massachusetts, United States). Ribonucleic acid (RNA) was extracted from feces and microcosm pellets at C1 and C7 using the RNeasy® kit (Qiagen) with modifications. Briefly, samples were mechanically lysed in the presence of guanidine-thiocyanate and ß-mercaptoethanol using the FastPrep® (four times for 30s at 5 m/S) (MP Biomedicals™, California, United States). RNA quality and concentration were assessed with Nanodrop and Bioanalyzer (Agilent, RNA integrity score >8). The RNA samples were reverse transcribed using a High-Capacity cDNA Reverse Transcription Kit (Applied Biosystems, Massachusetts, United States).

### 16S ribosomal ribonucleic acid gene sequencing and data analysis

The V3–V4 region (341F = CCTACGGGNGGCWGCAG, 805R = GACTACHVGGGTATCTAATCC [17]) of the 16S ribosomal RNA (rRNA) gene was sequenced using the Illumina Miseq technology by the Integrated Microbiome Resource at Dalhousie University (Nova Scotia, Canada). The residual adapter and primer sequences were removed and the reads were checked for quality (minimum score ≥20) and length (minimum 200 base pairs) using the cutadapt tool [18]. Then SPAdes [19] was applied to correct known sequencing errors and then PEAR to merge the reads [20] to enhance data quality. Amplicon sequencing variants (ASVs) were determined using the Vsearch pipeline [21].

### Shotgun sequencing and data analysis

Shotgun sequencing of C7 samples were performed by Novogene (Cambridge, United Kingdom). Briefly, genomic DNA was fragmented, a sequencing library was prepared, and subjected to paired-end (PE-150 bp) Illumina sequencing. Low-quality reads and adapters were removed with Fastp (v 0.23.1). Host sequences were removed using Bowtie2 (v 2.2.4) [22]. The clean data were assembled with MEGAHIT (v 1.2.9) [23], and gene prediction was performed using MetaGeneMark (v 2.1) [24]. Sequence redundancy was reduced using CD-HIT (v 4.5.8) [25] and clean data was mapped to a gene catalog with Bowtie2. Species annotation was performed with DIAMOND (v 2.1.6) [26] using the Micro_NR (v 2024.03) database and analyzed via the lowest common ancestor algorithm. Functional and resistance gene annotations were derived from the KEGG (v 2024.03) [27] and CARD databases [28].

### Real-time polymerase chain reaction of antimicrobial resistance genes

The quantitative polymerase chain reaction (qPCR) and real-time (RT)-qPCR were performed using the Power SybrGreen PCR Master Mix (Thermo Fisher Scientific) and a QuantStudio™ 3 RT thermocycler (Thermo Fisher Scientific) for the ARGs (Supplementary Table S1 [29–32]). The conditions were 50°C for 2 min, 95°C for 10 min, 40 cycles of 95°C for 15 sec, and 56°C for 1 min, followed by a melting curve analysis. The abundances of the genes were calculated based on calibration curves. Briefly, the amplified gene products were purified with MinElute® PCR purification kit (Qiagen), cloned into TOPO®TA (Thermo Fisher Scientific) vectors, and transformed using TOP10-competent *Escherichia coli* cells. The plasmids from the competent cells were extracted using the NucleoSpin®Plasmid kit (Macherey Nagel, Düren, Germany). The calibration curves were built by serially diluting the extracted plasmids (0.1 to 0.000001 ng) and converting the CT values to copy numbers.

### Metabolome analysis

The global metabolome of the filtered supernatants of CBZ and DMSO-treated microcosms at C0, C1, and C7 were performed by Metabolon Inc. using ultra high-performance liquid chromatography-tandem mass spectroscopy (UPLC-MS/MS). Metabolites were recovered by methanol precipitation of the proteins via centrifugation. The samples were analyzed with four different methods: two separate reverse phases (RP)/UPLC-MS/MS methods with positive ion mode electrospray ionization (ESI), RP/UPLC-MS/MS with negative ion mode ESI, and HILIC/UPLC-MS/MS with negative ion mode ESI. The peaks identified were compared to a library of purified standards or recurrent unknown entities. Each peak was quantified using the under-curve area and normalized to correct variation from the instruments used.

### Statistical methods

The data analyses were performed using RStudio (v 2022.12.0) and Prism GraphPad v9. The graphical illustrations for alpha diversity, taxa composition, principal component analysis (PCA), principal coordinate analysis (PCoA), taxa abundances, and heatmaps were obtained via phyloseq (v 1.46.0), ggplot2 (v 2.5.0), and factoextra (v 1.0.7) [33, 34]. The hierarchical clustering was made using the pvclust package (v 2.2-0) with Canberra distance and the ward.D2 clustering method [35]. The pvclust package furnishes two different *P*-values: approximately unbiased (AU) *P*-value and bootstrap probability (BP) *P*-value represented in percentage (significative if *P* > 95%). The statistical differences of the medians among the groups were assessed with the Mann–Whitney U-test, Multiple Wilcoxon test, the paired *t*-test, or analysis of variance (ANOVA) analysis, and false discovery rate (FDR) corrections were applied. Spearman’s correlation coefficients were calculated to assess the associations between metabolites, species, and genes.

## Results

### The semi-continuous cultivation strategy preserved the inter-individual variation among the fecal microbiomes

**Figure 2:**
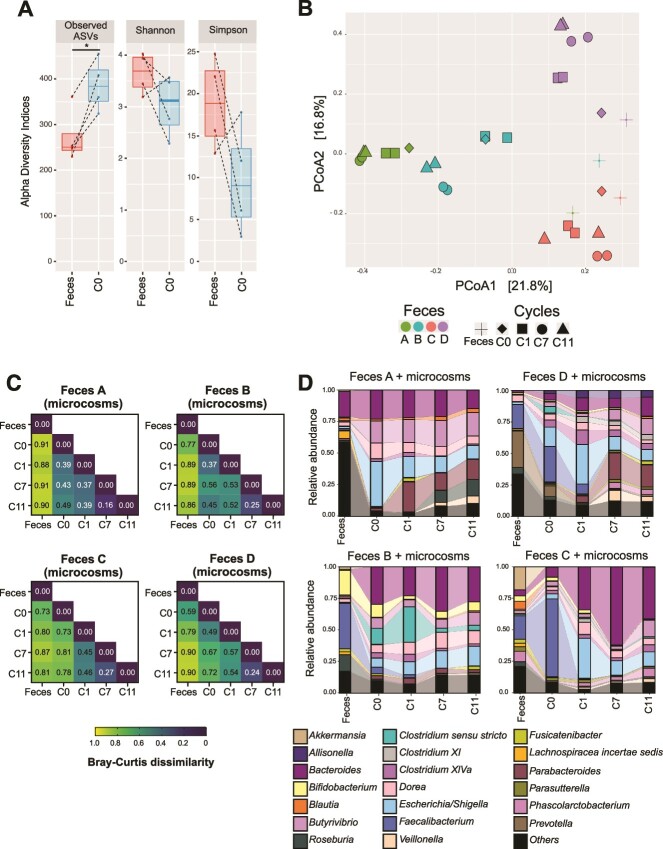
Semi-continuous cultivation strategy and feces (inoculum) composition modulated the microcosm structure and composition. Longitudinal microbiome structure of the feces-derived microcosms without ASM exposure. (A) Alpha diversity indices calculated for the microbiome of feces and the 24 h feces-derived C0 microcosms. ANOVA test ^*^P < .05. (B) PCoA of feces and the microcosms cultivated daily for 11 cycles. (C) Bray–Curtis dissimilarity matrix between the feces and feces-derived microcosms for each feces type (A, B, C, and D). (D) The relative abundance of the top 20 genera of the feces and the microcosms at C0, C1, C7, and C11 (41.7%–97.1% of the fecal microbiome composition was retained within the microcosms depending on the fecal inoculum type).

We investigated the stability and dynamics of feces-derived microcosms using a semi-continuous cultivation approach that relies on daily transfer of 2.5% of the previous culture to fresh media for 12 24-h cycles (C0–C11). For this analysis, we exclusively focused on microcosms without any ASMs exposure. Compared to the feces, 24 h microcosms (C0) had greater microbial richness based on the number of ASVs (ANOVA, *P* = .03) and lower Shannon and Simpson diversity (not statistically significant due to inherent inter-variability of the fecal inoculums, ANOVA *P* > .05; Fig. 2A). These metrics indicated that the 24-h cultivation in a highly nutrient-rich medium may lead to uneven growth of microbial community members and reduce the community evenness.

Next, we evaluated the longitudinal development of microbial communities via PCoA as shown in Fig. 2B. The PCoA demonstrated that feces (A, B, C, and D) and their microcosms at C0, C1, and C7 clustered mainly based on the fecal composition and dominated by different taxa. For example, B microcosms were associated with *Clostridium XIVa* ASVs and C microcosms were associated with *Bacteroides* ASVs (Supplementary Fig. S1). We also observed a drift in the microbiome structure through the cultivation cycles. The microcosms at C7 and C11 were more similar to each other (Bray–Curtis dissimilarity index of 0.23) compared to C0 or C1 (Bray–Curtis dissimilarity index of 0.62 and 0.47; Fig. 2C and Supplementary Fig. S2).

We also examined the microbiome composition of the feces and their microcosms (C0, C1, C7, and C11). Compared to the feces, C0 microcosms were enriched in *Escherichia/Shigella*, *Butyrivibrio*, and *Dorea,* and depleted in the *Lachnospiraceae incertae sedis* (Mann–Whitney U-test, *P* < .05; Fig. 2D, Supplementary Fig. S3 and Supplementary Table S2). C0 microcosms were also enriched in *Bacteroides* and *Clostridium XIVa* and depleted in the *Akkermansia*, *Bifidobacterium*, *Blautia*, and *Roseburia* (Mann–Whitney U-test, 0.1 > *P* > .05). Besides global trends, there were feces-dependent trends. For example, the relative abundance of *Faecalibacterium* increased only in C and D microcosms (from 19% for both to 62% and 28%, respectively) and decreased in B microcosms (from 31% to 5%).

From C0 to C1, microbiome composition changed (Fig. 2D). For example, the relative abundance of *Faecalibacterium* decreased from C0 to C1 in all cultures by 21%. In contrast, both *Escherichia/Shigella* and *Parabacteroides* relative abundance increased from C0 to C1 by 7% (Supplementary Table S2). The composition of microcosms continued to change from C1 to C7 and were more similar between C7 and C11 (Fig. 2D). For example, *Escherichia/Shigella* relative abundance decreased by 10% and remained at similar levels at C11. Overall, the combination of the serial transfer approach and the feces type drove the development of the microcosms after seven cycles of cultivation, therefore, did not generate compositionally similar microcosms.

### Carbamazepine had the largest impact on the microbiome composition among the three antiseizure medications

**Figure 3:**
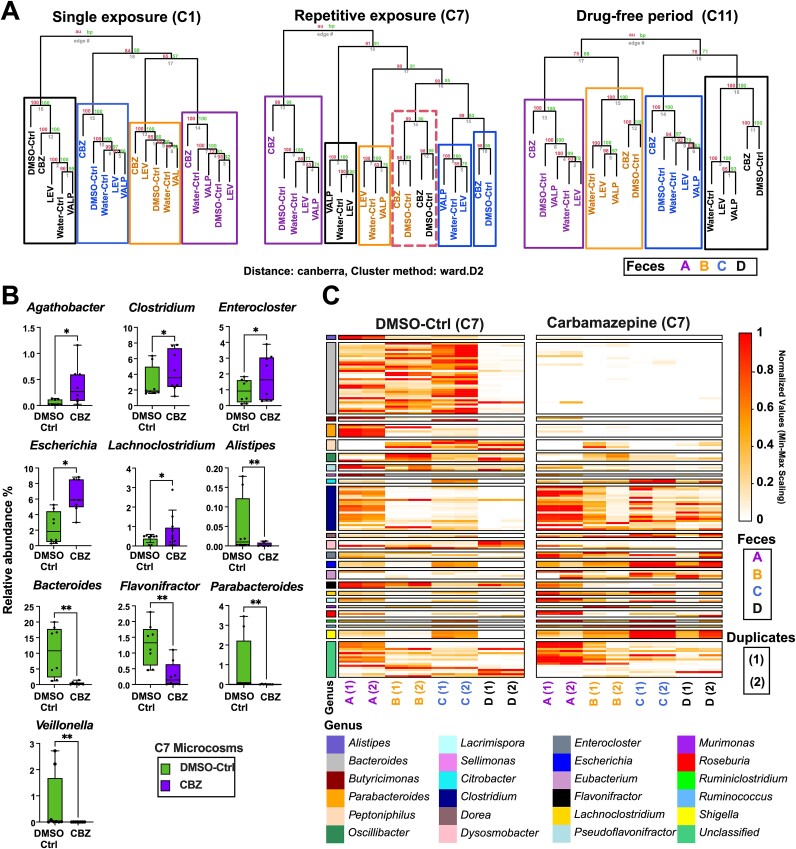
CBZ had the strongest impact on the microbial community structure. (A) Hierarchical clustering analysis of the microcosms exposed to CBZ, LEV, VALP, or drug-free controls (Water-Ctrl or DMSO-Ctrl) at single exposure (C1), repetitive exposure (C7), and after four drug-free cultivation (C11) period based on 16S rRNA gene sequencing. The analysis was based on the Canberra distance and War.D2 clustering method. The bootstrap values were calculated using two methods: AU *P*-value (the numbers on the left side of the dendrograms) made using multiscale bootstrap resampling and the BP value (the numbers on the right side of the dendograms) created using normal bootstrap resampling. (B) The relative abundance of five significantly enriched and five depleted genera at C7 (after repetitive exposure) based on shotgun metagenome sequencing. Multiple Wilcoxon tests for the comparison between CBZ and DMSO-Ctrl. ^*^P ≤ .05, ^*^^*^P ≤ .01. FDR discovery: Q > 0.05 (C) Heatmap of species that were significantly different (multiple Wilcoxon test, *P* ≤ .015) between CBZ and DMSO-Ctrl at C7 (after repetitive exposure). FDR discovery, q > 0.05.

To assess the repetitive exposure impacts of the ASMs on the microbiome, the microcosms were daily exposed to CBZ, VALP, or LEV for seven cycles (C1 to C7). Based on Ward’s hierarchical clustering method, at C1 which represents single exposure, all microcosms were significantly clustered based on feces type rather than the ASM type (AU and BP = 100; Fig. 3A). However, in each cluster, CBZ-exposed microcosms were distinctly separate from those exposed to LEV, VALP, or from the drug-free controls (DMSO-Ctrl and Water-Ctrl). At C7, representing repetitive exposure, the influence of CBZ on microcosm structure became more prominent, and feces type became less determinant as CBZ (along with DMSO-Ctrl) formed a cluster encompassing B, C, D microcosms (AU and BP > 95). Within this cluster, CBZ and DMSO-Ctrl microcosms formed sub-clusters.

Subsequently, we compared the composition of CBZ and DMSO-Ctrl at C7 through shotgun sequencing. The relative abundance of 83 genera differed significantly between the CBZ and DMSO-Ctrl (multiple Wilcoxon test, *P* < .05; Supplementary Table S3). In the CBZ, 38 genera were significantly more abundant compared to DMSO-Ctrl including *Agathobacter*, *Clostridium*, *Enterocloster*, *Escherichia*, and *Lachnoclostridium* (multiple Wilcoxon test, *P* < .05; Fig. 3B) and 45 genera were lower including *Bacteroides*, *Parabacteroides*, *Flavonifractor*, *Alistipes*, and *Veillonella* (multiple Wilcoxon test, *P* < .01). The relative abundances of 310 species (belonging to 103 different genera) were significantly different between the CBZ and DMSO-Ctrl (Multiple Wilcoxon test, *P* < .05) (Supplementary Table S4). Several *Bacteroides*, *Parabacteroides*, *Peptoniphilus* species (i.e. *Bacteroides thetaiotaomicron*, *Parabacteroides distasonis*, and *Peptoniphilus grossensis*) were significantly less in CBZ compared to DMSO-Ctrl and several *Clostridium* species *(*i.e. *Clostridium sp. Marseille-P7770*) were significantly more in the CBZ (multiple Wilcoxon test, *P* < .05; Fig. 3C and Supplementary Table S4). Additionally, CBZ samples had significantly higher abundance of *Dorea formicigenerans*, *Murimonas intestini*, or *Roseburia intestinalis* compared to DMSO-Ctrl and significantly lower abundance of *Faecalibacterium sp. Marseille-Q3530* (Multiple Wilcoxon test, *P* < .05; Supplementary Table S4).

We also compared the VALP and Water-Ctrl at C7 (based on 16S rRNA gene sequencing) and observed significantly lower abundance of *Escherichia*/*Shigella (*Mann–Whitney U-test, *P* = .02), lower abundance of *Anaerobacter*, and *Isobaculum*, and higher abundance of *Melissococcus*, and *Vagococcus (*Mann–Whitney U-test, *P* < .05). At C7, no genera were significantly different between the LEV and Water-Ctrl (Mann–Whitney U-test, *P* > .05).

Next, we evaluated the resilience of microcosms by refraining the ASMs for four cultivation cycles (C8 to C11). The C11 microcosms clustered more similarly to C1 microcosms than C7, with feces type as inoculum source emerged as the predominant factor shaping the communities, overshadowing the previous ASM exposure history. Once the CBZ was refrained from C8 to C11, several taxa that were depleted during the repetitive exposure cycles including *Clostridium XlVa*, *Flavonifractor*, or *Bacteroides* increased to reach similar levels of the controls (Water-Ctrl and DMSO-Ctrl; Supplementary Fig. S4). In contrast, refraining CBZ depleted *Murimonas*, *L. incertae sedis*, and *Escherichia/Shigella* that were either enriched or not impacted during the repetitive exposure cycles (Supplementary Fig. S4). Overall, repetitive exposure to CBZ resulted in a profound impact on community composition, and this effect was partially overcome by four cycles of the drug-free recovery period.

### Repetitive carbamazepine exposure increased the abundance and expression of antimicrobial resistance genes

**Figure 4:**
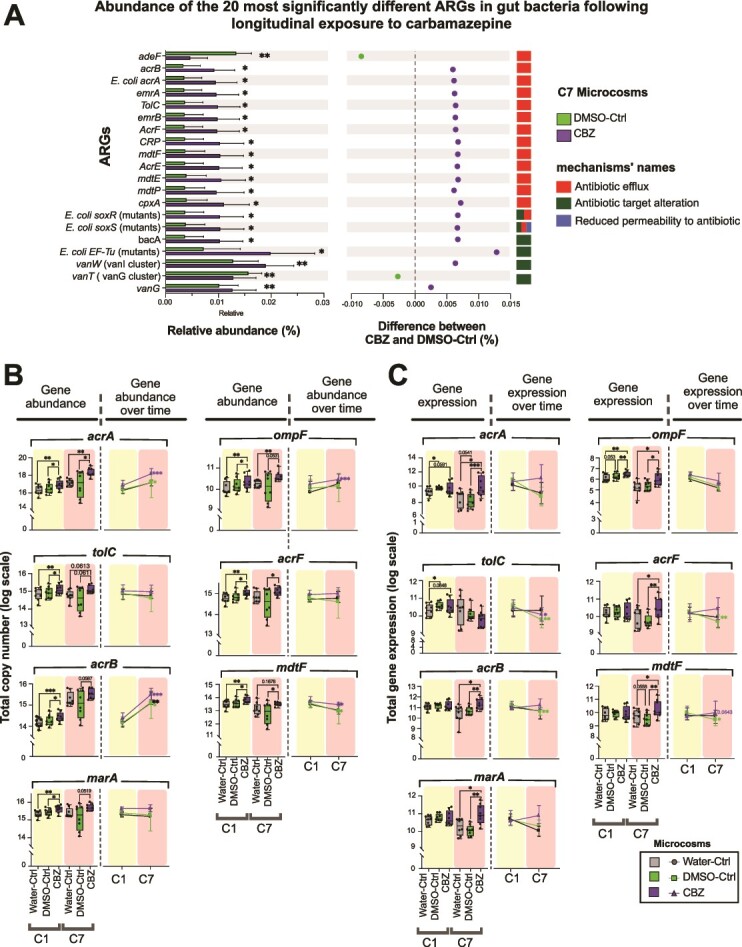
Repetitive exposure to CBZ increased the abundance and expression of ARGs in the microcosms. (A) Relative abundance of the 20 most abundant and significantly different genes involved in antibiotic resistance based on shotgun metagenomic sequencing, between CBZ and DMSO-Ctrl (multiple Wilcoxon test, *P* ≤ .05). FDR discovery, q < 0.05. (B) Abundance of seven ARGs in the CBZ, Water-Ctrl, and DMSO-Ctrl at C1 and C7 based on q-PCR. Paired *t*-test, ^*^P ≤ .05, ^*^^*^P ≤ .01, ^*^^*^^*^P ≤ .001. The right panel depicts the seven AMR genes at C1 and C7. Statistics: paired *t*-test, ^*^P ≤ .05, ^*^^*^P ≤ .01, ^*^^*^^*^P ≤ .001. (C) Expression levels of seven AMR genes in the CBZ, Water-Ctrl, and DMSO-Ctrl at C1 and C7 based on RT-PCR. Paired *t*-test, ^*^P ≤ .05, ^*^^*^P ≤ .01, ^*^^*^^*^P ≤ .001. The right panels represent AMR gene expression at C1 and C7. Paired *t*-test, ^*^P ≤ .05, ^*^^*^P ≤ .01, ^*^^*^^*^P ≤ .001. FDR discovery, 0.087 ≥ q ≥ 0.0392.

We assessed the impact of repetitive CBZ exposure on the antimicrobial resistance (AMR) mechanisms in CBZ and DMSO-Ctrl. Based on shotgun metagenomic sequencing, 152 ARGs were detected in C7 samples, and 69 ARGs, associated mostly with antibiotic target alteration and antibiotic efflux, were significantly different between the CBZ and DMSO-Ctrl (Multiple Wilcoxon test, *P* < .05; Fig. 4A, Supplementary Fig. S5, and Supplementary Table S5). Notably, the relative abundances of several antibiotic efflux system genes, including *acrB*, *TolC*, *mdtF*, and *E. coli acrA*, were significantly higher in the CBZ compared to the DMSO-Ctrl (multiple Wilcoxon test, *P* < .05, q < 0.05; Fig. 4A). Subsequently, we quantified the abundance and expression levels of several ARGs at C1 and C7 that were over-represented in the CBZ. At C1, *acrA*, *tolC*, *acrB*, *marA*, *ompF*, *acrF*, and *mdtF* genes in CBZ were significantly higher in CBZ compared to the DMSO-Ctrl and Water-Ctrl (paired *t*-test, *P* < .05; Fig. 4B). However, at C7, only the abundance of *acrA*, *acrF*, and *mdtF* were higher in CBZ compared to the DMSO-Ctrl (paired *t*-test, *P* < .05).

Unlike the gene abundance profiles at C1, the expression levels of ARGs at C1 (besides *tolC* and *ompF*) were not significantly different between CBZ and DMSO-Ctrl. However, at C7, the expression of *acrA*, *acrB*, *marA*, *ompF acrF*, and *mdtF* genes were significantly higher in the CBZ compared to DMSO-Ctrl and Water-Ctrl (paired *t*-test, *P* < .001; Fig. 4C). Our results indicated that single exposure to CBZ increased the abundance of several ARGs; however, the expression of most of those genes (especially *acrB*, *marA*, *acrF*, and *mdtF*) were only induced after repetitive exposure.

### Carbamazepine impacted the energy metabolism and the metabolism of glutamate, spermidine, and vitamin B6

**Figure 5:**
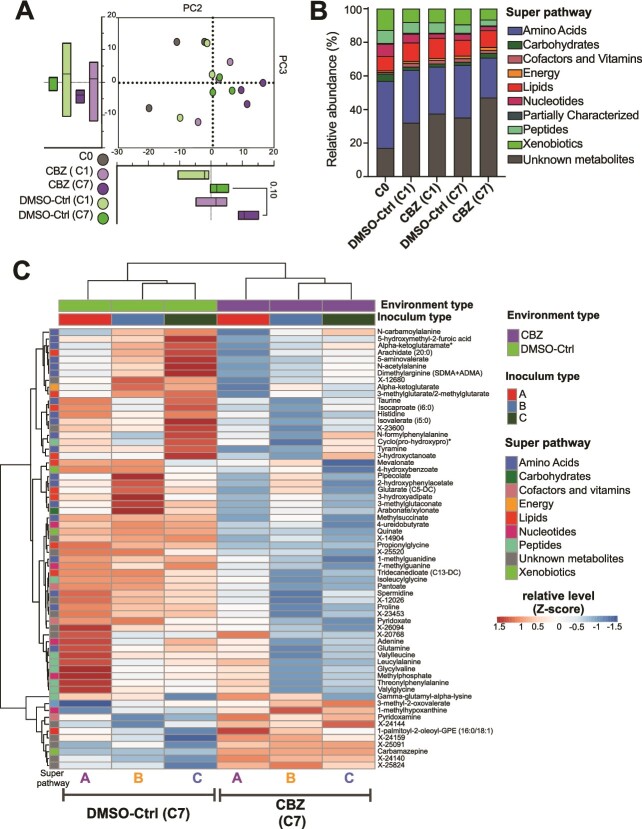
Changes in the microcosm structure due to CBZ exposure were reflected on the TCA cycle and metabolism of glutamate, spermidine, and vitamin B6. (A) PCA of the metabolomes of the C0 microcosms as well as the CBZ (CBZ-exposed) and DMSO-Ctrl (control) microcosms at C1 (single-exposure) and C7 (repetitive-exposure). The bar plot represents the coordinates of the PC2 and PC3. No statistical difference between CBZ and DMSO-Ctrl (Mann–Whitney U-test, *P* > .05). (B) Distribution of the metabolites within the super pathways belonging to C0 (24 h feces-derived culture), CBZ and DMSO-Ctrl microcosms at C1 and C7. (C) Metabolites that significantly differed (paired *t*-test, *P* ≤ .05) between the CBZ and DMSO-Ctrl microcosms at C7.

To identify changes in microbial metabolism due to the repetitive CBZ exposure, we analyzed the global metabolome of the supernatants of CBZ and DMSO-Ctrl derived from 3 feces (A, B, and C) at C0, C1, and C7. 626 metabolites were identified belonging to the main super-pathways: amino acid (31.4%), lipid (10.7%), xenobiotic (9.1%), peptide (5.7%), and nucleotide (3.9%). PCA showed that the CBZ treatment altered the metabolome, and the effect size was larger at C7 than C1 (Mann–Whitney U-test, *P* = .1), reflecting the changes in the microbial community structure (Fig. 5A). CBZ had a lower abundance of amino acids and a greater abundance of unknown metabolites compared to DMSO-Ctrl (Fig. 5B). These differences were greater at C7 than C1, although not statistically significant (Supplementary Fig. S6).

**Figure 6:**
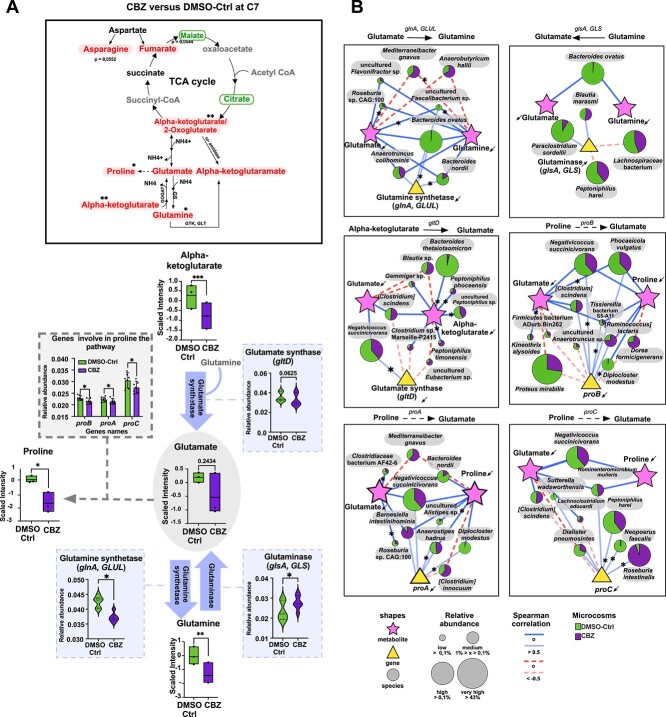
Longitudinal exposure to CBZ impacted the TCA cycle, glutamate and proline metabolisms. (A) Representation of the TCA cycle and glutamate metabolism and their associated genes. The metabolites higher in CBZ compared to DMSO-Ctrl were highlighted by circles, and those lower in CBZ compared to DMSO-Ctrl were represented by shaded background. The metabolites that do not change between CBZ and DMSO-Ctrl were represented in black, and the undetected ones in grey. Box plots of alpha-ketoglutarate, glutamate, glutamine, and proline in CBZ and DMSO-Ctrl at C7. The significantly different metabolites between CBZ and DMSO-Ctrl based on paired *t*-test, ^*^P ≤ .05, ^*^^*^P ≤ .01, ^*^^*^^*^P ≤ .001 (FDR discovery, q > 0.05). The box plots represent the relative abundance of genes significantly different between CBZ and DMSO-Ctrl at C7. Multiple Wilcoxon test ^*^P ≤ .05, ^*^^*^P ≤ .01, ^*^^*^^*^P ≤ .001. FDR discovery, q > 0.05. (B) Correlation network between glutamate pathway metabolites, related genes, and the species linked to those genes based on shotgun metagenomics. Each panel represents one reaction in the glutamate pathway, showing the relationships between the metabolites, the genes involved in the metabolism, and bacterial species containing the genes. Metabolites are depicted as stars, genes as triangles, and bacterial species as circles. The dashed lines indicate negative correlations, while the solid lines represent positive correlations. The bacterial species shown exhibited either a minimum of two correlations with a value >0.5 or <−0.5, or one correlation with a statistically significant correlation (Spearman correlation, ^*^P ≤ .05, ^*^^*^P ≤ .01). The pie charts demonstrate percentage of the indicated bacterial species in the CBZ (in purple) or the DMSO-Ctrl (in green). The size of each pie chart corresponds to the overall abundance of bacterial species.

At C7, 52 metabolites were depleted, and 11 were enriched in the CBZ compared to DMSO-Ctrl (paired *t*-test, *P* < .05; Fig. 5C). More than half of the depleted metabolites in CBZ belonged to peptide and amino acid pathways, including glutamine, proline, histidine, or leucyl-alanine. Interestingly, several metabolites involved in the glutamate metabolism and TCA cycle were co-depleted in CBZ (Fig. 6A, Supplementary Fig. S7). For example, alpha-ketoglutarate and fumarate were at lower abundance (paired *t*-test, *P* < .05, respectively) whereas malate and citrate were at higher abundance in CBZ compared to DMSO-Ctrl (Fig. 6A). Besides its role in the TCA cycle, alpha-ketoglutarate can be converted into glutamate via glutamate synthetase enzyme (GOGAT). The *glutD* gene encoding GOGAT, was at lower abundance in CBZ compared to DMSO-Ctrl (multiple Wilcoxon test, *P* = .0625). The interconvertible glutamate and glutamine were both lower in CBZ. The conversion of glutamate to glutamine is catalyzed by the glutamine synthetase, encoded by the *glnA* and *GLUL* genes, which were significantly lower in CBZ compared to DMSO-Ctrl (multiple Wilcoxon test, *P* = .0312; Fig. 6A). In contrast, the relative abundance of *glsA* encoding for glutaminase that converts glutamine to glutamate, was higher in CBZ than in DMSO-Ctrl (Multiple Wilcoxon test, *P* = .0312). Alpha-ketoglutaramate, a precursor of alpha-ketoglutarate and metabolite of glutamine, was also significantly depleted in CBZ (paired *t*-test, *P* = .03). Additionally, proline which can be produced from glutamate through the catalytic activity of several enzymes including γ-glutamyl kinase, γ-glutamyl phosphate reductase and pyrroline-5-carboxylate reductase encoded by *proB*, *proA*, and *proC*, respectively. In CBZ both proline (paired *t*-test, *P* = .02) and the genes involved in its production were depleted (multiple Wilcoxon test, *P* < .05; Fig. 6A).

Even though butyrate levels were not significantly different between CBZ and DMSO-Ctrl, the relative abundance of several enzymes involved in the butyrate pathway varied between CBZ and DMSO-Ctrl. Specifically, enzymes converting acetyl-CoA to crotonyl-CoA were significantly higher in CBZ (*P* < .05), while those converting crotonyl-CoA to butyrate were lower, though not statistically significant (Supplementary Fig. S8).

To link microbial communities to glutamate pathway metabolites, we conducted a Spearman correlation analysis between metabolites, pathway genes, and species harboring the pathway genes (Fig. 6B; Supplementary Table S6). Glutamate and glutamine positively correlated with several species, including *uncultured Faecalibacterium sp.* [Spearman’s rank coefficient (rs) > 0.8], *Bacteroides nordii* (rs = 0.71 and 0.66, respectively), and *Bacteroides ovatus* (rs = 0.71 and 0.66, respectively). Notably, the depletion of *B. ovatus* in CBZ was linked to decreased levels of *glnA* and glutamate. Additionally, alpha-ketoglutarate showed a strong positive correlation with *B. thetaiotaomicron* (rs = 0.88, *P* = .03). Proline levels positively associated with genes involved in proline production (*proA*, *proB*, and *proC*) and species harboring these genes (i.e. *Negativicoccus succinicivorans or B. nordii*), all of which were significantly lower in CBZ.

**Figure 7:**
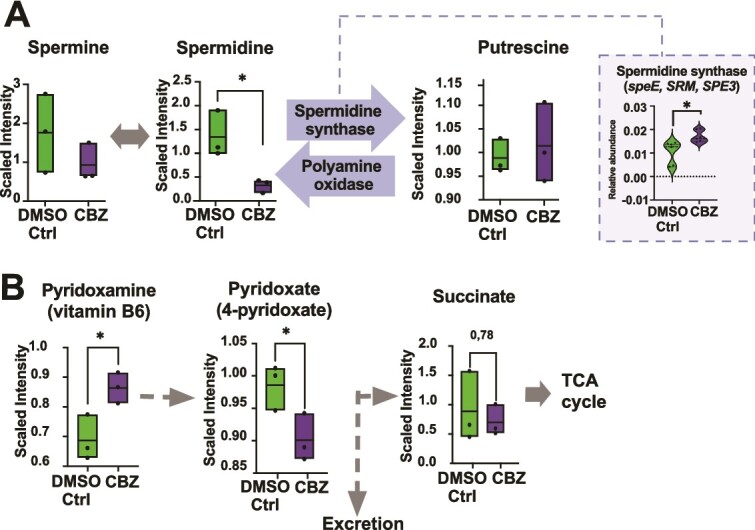
CBZ exposure altered the spermidine and vitamin B6 metabolisms. Representation of the spermidine (A) and vitamin B6 (B) metabolites and associated genes that significantly differed between CBZ and DMSO-Ctrl at C7. The box plot compares the abundance of the metabolites in the CBZ and DMSO-Ctrl. Paired *t*-test, ^*^P ≤ .05, ^*^^*^P ≤ .01, ^*^^*^^*^P ≤ .001. (FDR discovery, q > 0.05). The box plot highlighted in the dashed rectangle represents the genes significantly different between CBZ and DMSO-Ctrl. Multiple Wilcoxon test (^*^P ≤ .05, ^*^^*^P ≤ .01, ^*^^*^^*^P ≤ .001). FDR discovery, q > 0.05.

Repetitive CBZ exposure also impacted polyamine metabolism. At C7, spermidine was significantly lower in CBZ compared to DMSO-Ctrl (paired *t*-test, *P* = .03; Fig. 7A) while putrescine levels were higher and spermine levels were lower in CBZ compared to the DMSO-Ctrl (*P* > .05). The genes *speE*, *SRM*, and *SPE3*, encoding for spermidine synthase that can convert spermidine to putrescine, were significantly higher in the CBZ compared to the DMSO-Ctrl (multiple Wilcoxon test, *P* = .0312). Besides polyamines, vitamin B6 pathway was also impacted by the repetitive CBZ exposure. Pyridoxamine (a form of vitamin B6) and its downstream metabolic product pyridoxate were significantly higher and lower, respectively, in the CBZ compared to DMSO-Ctrl (paired *t*-test, *P* = .018 and .046, respectively). Pyridoxate can either be excreted or transformed into succinate (another TCA cycle metabolite; Fig. 7B).

## Discussion

Our study demonstrates that ASMs may longitudinally modify gut microbiome and metabolome dynamics, and even though these effects show interpersonal characteristics, some of them may induce global shifts, including a reduction of commensals, increase of ARGs and depletion of dipeptides which may adversely impact the homeostasis and health of the host.

Even though earlier studies investigated the impact of ASMs on commensal bacteria [9, 11], they characterized single-exposure effects on microbial growth in monocultures. An important implication of our study is that repetitive exposure to CBZ has a cumulative effect on gut microbiome dynamics, an effect that could be easily overlooked by single-exposure studies. The complex gut ecosystem of children with epilepsy is chronically exposed to ASMs as daily treatment lasts for several years. Repetitive CBZ exposure depleted key gut bacteria, such as *B. thetaiotaomicron*, which produces beneficial metabolites through mucin degradation [36] and supports the development of colonic goblet cells [37]. Additionally, CBZ affected several butyrate producers: *Faecalibacterium sp.* and *Alistipes sp.* were depleted, *R. intestinalis* and *Clostridium* sp. were enriched, along with slight reductions in several butyrate production enzymes [38]. Butyrate concentration remained unchanged potentially due to the inherent functional redundancy in microbial communities.

A limitation is that CBZ, insoluble in water, required dissolution in DMSO. Despite DMSO concentration being <1%, a level previously reported not to affect monoculture growth [11], we demonstrated it has an impact on complex microbial community assembly. Another aspect of our study involved the examination of the resilience of microbial communities following repeated exposure to ASMs. A partial recovery of microbial composition after a drug-free period indicated that some community members such as *Bacteroides sp.* can bounce back from the adverse microbial effects of CBZ.

Previous studies revealed an increase in the transmission of ARGs in wastewater ecosystems due to the accumulation of CBZ [13–15]. Therefore, in our study, we purposely investigated AMR profiles and, for the first time, demonstrated an increase in the abundance and expression of ARGs involved in different mechanisms such as efflux pump, target alteration, antibiotic inactivation, or target protection in feces-derived cultures repetitively exposed to CBZ. For example, an increase in the abundance and expression of *acrA and acrB* genes, both belonging to a periplasmic adaptor protein from the multidrug efflux pump AcrAB-TolC, due to repetitive CBZ exposure may facilitate excretion of antibiotics in Gram-negative bacteria [39]. Our results suggest an adaptation-based selection process, as expression of some AMR genes increased only after consistent repetitive exposure. CBZ increases the permeability of the cellular membranes [14], therefore may select for microorganisms that are equipped with more resistant membrane properties and expressing genes to preserve membrane integrity.

Our integrative analysis highlighted that shifts observed in microbial dynamics were reflected in the metabolome. This is consistent with findings from a recent study on rats treated with valproate and depletion of certain SCFAs [10]. In our in-vitro study, depletion of the commensals due to CBZ treatment led to a decrease in key metabolites involved in energy, amino acid, and vitamin metabolisms. Depletion of alpha-ketoglutarate, a precursor of non-essential amino acids, along with its derivatives, glutamine, and proline and vitamin B6 metabolites might influence microbial dynamics in the gut. For example, changes in the glutamate metabolism have been correlated with a higher abundance of *Eggerthella lenta* and *Clostridium botulinum* in children with Autism Spectrum Disorder [40]. Additionally, glutamine and pyridoxamine improve the resilience of microorganisms to stressors [41, 42] and vitamin B6 metabolites are essential for microbial amino acid metabolism [43]. Therefore, depletion of these metabolites may increase vulnerability of key microbial species to CBZ.

The shifts in microbial glutamine and vitamin metabolisms may also influence host health. Glutamine is a non-essential amino acid that maintains the gut barrier function by enabling cell proliferation and differentiation as well as regulating paracellular permeability [44]. Additionally, glutamine is the precursor of two neurotransmitters, gamma-aminobutyric acid (GABA) and glutamate, that are involved in modulation of seizures, hence critical in maintenance of the neurological health [45, 46]. Some commensals, such as *B. ovatus*, can produce glutamate from glutamine [47]. A study on mice demonstrated that *B. ovatus* colonization enhanced the abundance of neurotransmitters glutamate and GABA [48]. In our CBZ-exposed microcosms, both neurotransmitters were depleted alongside a reduction in *B. ovatus*. Although CBZ reduces the levels of these microbially produced neuromodulatory metabolites, the potential effects on the brain remain unclear due to limitations in regard to blood–brain barrier research.

Spermidine is another neuromodulatory metabolite, a polyamine that can be produced by the microbiota, including *Bacteroides* sp., and decreased in CBZ-exposed microcosms paralleling changes in the relative abundance of *Bacteroides* species. It has several important roles in the integrity of blood brain barrier [49] or the gut epithelial barrier [50], and it was shown to have pro-epileptic effects by shortening the seizure latency using pentylenetetrazol in rats [51]. Another important metabolite in the management of seizures is vitamin B6. In our microcosms, CBZ increased vitamin B6 and depleted its byproduct, pyridoxal, suggesting a reduction in bacteria utilizing vitamin B6. A deficiency and dependency of vitamin B6 is associated with epileptic seizures [52], and supplementation of B6 is recommended in its therapy [53]. The human body cannot synthesize this vitamin; they rely on dietary intake or vitamin-producer bacteria (i.e. *B. fragilis*) [43]. Therefore, an increase in vitamin B6 reserves and depletion of spermidine in microbial communities due to CBZ may indicate a potential added benefit to the treatment.

In conclusion, our study provides insights into the direct effects of commonly prescribed ASMs on gut microbiome structure and function as an evolutionary process. As most drug-microbiome studies focus on acute effects, our study fills a gap in the knowledge by addressing repetitive effects. Moreover, our experiment design with four biological replicates highlighted the inter-individual variability in the drug-microbiome interactions. We demonstrated that repetitive exposure to a commonly used ASM, CBZ, impacted the gut microbiome by shifting the composition and depleting essential microbial metabolites. Moreover, our findings indicate a potential increase in the gut resistome of children who are under chronic CBZ treatment.

## Supplementary Material

Supplementary_Table_S1_and_Supplementary_Figures_S1-S7_ycae123

Supplementary_Tables_S2-S7_ycae123

## Data Availability

The 16S rRNA gene reads are publicly available from the National Center for Biotechnology Information Sequence Read Archive under the Bioproject accession number PRJNA1072074. The other datasets generated are available from the corresponding author on request.
