# Supplementary material for: Longitudinal exposure to antiseizure medications shape gut-derived microbiome, resistome, and metabolome landscape
Source: ISME Commun. 2024 Oct 18;4(1):ycae123. doi: 10.1093/ismeco/ycae123 (PMC11544314; doi:10.1093/ismeco/ycae123)
Supplement: Supplementary_Table_S1_and_Supplementary_Figures_S1-S7_ycae123 [file supplementary_table_s1_and_supplementary_figures_s1-s7_ycae123.docx]

**Supplementary Table S1.** Primer sequences of the selected genes for the qPCR and RT-qPCR assays.

| ***Gene name*** | ***Gene*** | ***Forward*** | ***Reverse*** | ***Size*** | ***Reference*** |
| --- | --- | --- | --- | --- | --- |
| **Multidrug efflux pump subunit AcrA** | *acrA* | CTCTCAGGCAGCTTAGCCCTAA | TGCAGAGGTTCAGTTTTGACTGTT | 107 | *(29)* |
| **Efflux pump membrane transporter** | *acrB* | GGTCGATTCCGTTCTCCGTTA | CTACCTGGAAGTAAACGTCATTGGT | 107 | *(29)* |
| **Outer membrane protein TolC** | *tolC* | AAGCCGAAAAACGCAACCT | CAGAGTCGGTAAGTGACCATC | 100 | *(29)* |
| **Multidrug export protein AcrF** | *acrF* | TAGCAATTTCCTTTGTGGTT | CCTTTACCCTCTTTCTCCAT | 247 | *(30)* |
| **Multiple antibiotic resistance A protein** | *marA* | CATAGCATTTTGGACTGGAT | TACTTTCCTTCAGCTTTTGC | 187 | *(30)* |
| **Outer membrane porin F** | *ompF* | AAGTAGTAGGTTGCGCCCAC | AGTTCGATTTCGGTCTGCGT | *118* | *(31)* |
| **Multidrug resistance protein MdtF** | *mdtF* | TTACCGTCAGCGCTACCTATCC | GCCATCAAGCCCATTCATATTT | *95* | *(32)* |

**Supplementary Figure S1.** Principal component analysis representing microbiomes of fecal samples, feces-derived microcosms through daily serial-transfer based cultivation cycles (C0-C11) without any treatment**.** The ASVs represented on the left PCA are the 4 ASVs with the highest cos2 score.

**Supplementary Figure S2.** Bray-Curtis dissimilarity index between the microcosms cultivated for 11 cycles. A, B, C, and D represent the fecal samples as inoculum source of *in-vitro* microcosms through 12 cycles of serial-transfer based cultivation (C0, C1, C7, and C11).

**Supplementary Figure S3.** Relative abundance of genera that were enriched or depleted in 24h *in-vitro* feces-derived cultures (C0 microcosms) compared to the feces. The relative abundance of *Escherichia/Shigella*, *Butyrivibrio*, and *Dorea* significantly increased and *Lachnospiraceae incertae sedis* decreased in C0 microcosms compared to the feces. Mann-Whitney U test, *p < 0.05.

**Supplementary Figure S~~4~~.** Relative abundance of genera after repetitive carbamazepine exposure (C7) and drug-free period (C11). Mann-Whitney U test for comparing CBZ to DMSO-Ctrl or Water-Ctrl (*p ≤ 0.05, **p ≤ 0.01).


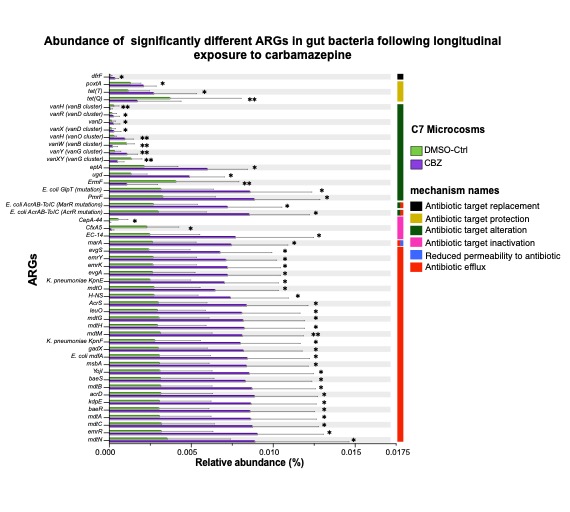


**Supplementary figure S5:** Relative abundance of significantly different ARGs after longitudinaly exposed to CBZ compared to DMSO-Ctrl Multiple Wilcoxon test (*p ≤ 0.05, **p ≤ 0.01, ***p ≤ 0.001).

**Supplementary Figure S6.** Relative abundance of the metabolites at the super-pathway level in CBZ or DMSO-Ctrl at C0, C1, and C7 for each feces-derived microcosm.

**Supplementary Figure S7.** Abundance of metabolites of the TCA cycle and glutamate metabolism in DMSO-Ctrl and CBZ at C7 (repetitive exposure). matched pairs t-test, *p ≤ 0.05, **p ≤ 0.01, ***p ≤ 0.001.

**~~~~**

**Supplementary Fig. S8** : Representation of the butyrate pathway. The box plot highlighted in blue represents the enzymes significantly different between CBZ (purple) and DMSO (green). Multiple Wilcoxon test (*p ≤ 0.05, **p ≤ 0.01, ***p ≤ 0.001). and The box plot with a dotted outline represent metabolites matched pairs t-test, *p ≤ 0.05, **p ≤ 0.01, ***p ≤ 0.001.
